# Building Capacity for Research on Community Doula Care: A Stakeholder-Engaged Process in California

**DOI:** 10.1007/s10995-023-03883-2

**Published:** 2024-01-24

**Authors:** Cassondra J. Marshall, Ashley Nguyen, Stephanie Arteaga, Erin Hubbard, Marna Armstead, Sayida Peprah-Wilson, Starr Britt, Monica R. McLemore, Anu Manchikanti Gomez

**Affiliations:** 1 School of Public Health, University of California, 2121 Berkeley Way #5302, Berkeley, CA 94720 USA; 2 Sexual Health and Reproductive Equity Program, School of Social Welfare, University of California, 120 Haviland Hall, Berkeley, CA 94720-7400 USA; 3 Department of Obstetrics, Gynecology, and Reproductive Sciences, University of California, 10490 Illinois St., San Francisco, CA 94143 USA; 4 SisterWeb San Francisco Community Doula Network, 1912 Keith St., San Francisco, CA 94124 USA; 5 Diversity Uplifts, Inc., 6371 Haven Ave., Suite 3, #265, Rancho Cucamonga, CA 91737 USA; 6 Starr Public Relations Group, 1630 Center St., Oakland, CA 94607 USA; 7 Department of Child, Family, and Population Health Nursing, School of Nursing, University of Washington, 1959 NE Pacific St., Seattle, WA 98195 USA

**Keywords:** Community doula, Doula care, Maternal health, Community engaged research, Research priorities

## Abstract

**Purpose:**

In an effort to address persistent inequities in maternal and infant health, policymakers and advocates have pushed to expand access to doula care. Several states, including California, now cover doula services through Medicaid. As coverage expands, research on the impact of doula care will likely increase. To develop best practices for research, it is critical to engage community doulas, clients, and other key stakeholders.

**Description:**

Our overarching goal was to build capacity for future doula- and client-centered research on community doula care. First, we established a Steering Committee with members from seven relevant stakeholder groups: community doulas, former or potential doula clients, clinicians, payers, advocates, researchers, and public health professionals. Second, we conducted a needs assessment to identify and understand stakeholders’ needs and values for research on community doula care. Findings from the needs assessment informed our third step, conducting a research prioritization to develop a shared research agenda related to community doula care with the Steering Committee. We adapted the Research Prioritization by Affected Communities protocol to guide this process, which resulted in a final list of 21 priority research questions. Lastly, we offered a training to increase capacity among community doulas to engage in research on community doula care.

**Assessment:**

Our findings provide direction for those interested in conducting research on doula care, as well as policymakers and funders.

**Conclusion:**

The findings of our stakeholder-engaged process provide a roadmap that will lead to equity-oriented research centering clients, doulas, and their communities.

## Significance

**What is Already Known on this Subject?:**

Doula care is often cited as a cost-effective intervention to improve maternal and infant health and health care experiences. However, research that focuses on the community-based model of doula care is limited.

**What this Study Adds?:**

This paper describes a stakeholder-engaged process that identified important understudied research questions about community doula care. Researchers can further refine these questions to conduct partnered research with multiple stakeholder groups, including community doulas, former or potential doula clients, clinicians, payers, advocates, and public health professionals. Further, this paper outlines best practices for research on community doula care.

## Purpose

In light of inequities in maternal and infant health in the United States (U.S.) (Howell, 2018; MacDorman et al., 2016; MacDorman & Mathews, 2011), doula care has often been cited as a cost-effective intervention (Kozhimannil et al., 2013; Kozhimannil, Hardeman, Kozhimannil et al., 2016) to improve health and health care experiences (Gruber et al., 2013; Kozhimannil, Vogelsang, Kozhimannil et al., 2016; Mallick et al., 2022; Thomas et al., 2017). Birth doulas are non-clinical, trained professionals who provide one-on-one educational, informational, physical, and spiritual support before, during, and after childbirth (Bey et al., 2019). Doulas can also provide support during miscarriage, stillbirth, and abortion (Goodman & Arora, 2022). Notably, doula support is associated with higher levels of respectful care in hospital settings, particularly for individuals who are Black, Asian/Pacific Islander, or insured by Medicaid (Mallick et al., 2022). This finding is particularly important given that Black women, who are disproportionately impacted by maternal mortality and morbidity, face structural racism and implicit bias in pregnancy-related care (Crear-Perry et al., 2021; Njoku et al., 2023).

Various models of doula care exist, including volunteer and/or paid hospital-based doula programs, doula collectives, community doula programs, and fee-based individual private doula care. Community doulas are members of the communities they serve and share the same backgrounds, cultures, and/or languages as their clients (Bey et al., 2019; Marshall et al., 2022). Community doulas often work with community-based doula programs, which mostly rely on public and private funding to provide low- or no-cost doula services to individuals who are more likely to experience adverse health outcomes (Bey et al., 2019; HealthConnect One, 2017). Community doulas may provide more home visits and referrals to social support services than traditional private doulas, who serve clients with more financial and social resources (Bey et al., 2019). The community-based model of doula care specifically focuses on reproductive justice and health inequities, making it a promising intervention to reduce racial disparities in maternal and infant health (Bey et al., 2019; Van Eijk et al., 2022). However, research that focuses on the community-based model of doula care is limited.

Nationwide, policymakers and advocates have pushed to expand access to doula care; several states now cover doula services through Medicaid (National Health Law Program, n.d.). California’s Medicaid program, Medi-Cal, covers nearly half of the 500,000 births each year in the state (Simon, 2020) and added doula services as a covered benefit in 2023 (Department of Health Care Services, n.d.). From 2019 to 2022, at least ten doula pilot programs launched in California to serve Black birthing people and Medi-Cal enrollees (Robles-Fradet & Chen, 2022). Health plans and the California Department of Public Health’s Perinatal Equity Initiative funded most pilots, many of which incorporated elements of community doula care (Robles-Fradet & Chen, 2022).

Research on the impact of doula care, including evaluations of different models, will likely increase as coverage of doula care increases. Recent reports have noted the need for future research on patient preferences for utilization of doula services, sustaining the doula workforce, and the impact of doula services on health outcomes and maternity care costs (Guenther et al., 2022; Knocke et al., 2022). To develop best practices for research, it is critical to engage community doulas, clients, and other key stakeholders. In this paper, we detail our stakeholder-engaged process to build capacity for client-centered, community-informed, and equity-focused research on doula care in California. Although our focus is California, we believe our process and the accompanying lessons learned provide important insights for conducting research on doula care more broadly.

## Description

Our overarching goal was to build capacity for future doula- and client-centered research on community doula care. Three university-based researchers invited three leaders of community-based doula organizations or initiatives in California to join the project leadership team. This project leadership team collaboratively developed four project objectives. Below we describe the steps we took to achieve each objective.

### Step 1: Establish and Engage Steering Committee

The leadership team identified seven relevant stakeholder groups as essential partners in future research on community doula care and, thus, to be engaged in the project. Stakeholder groups included community doulas, former or potential doula clients, clinicians (including physicians, midwives, and nurses), payers, advocates, researchers, and public health professionals. The leadership team established a Steering Committee with members from each stakeholder group to create infrastructure and help guide project activities by leveraging their professional networks to recruit members and inviting additional members based on suggestions from those initially selected. Community doulas and former doula clients were prioritized for membership given the importance of including their perspectives as individuals who have the lived experience of providing or receiving doula support.

The final Steering Committee included project leadership and consisted of community doulas (*n* = 4), former doula clients (*n* = 4), clinicians (*n* = 3), payers/health plan representatives (*n* = 2), advocates and policymakers (*n* = 2), researchers (*n* = 3), and public health professionals (*n* = 2). Members lived and worked in the San Francisco Bay Area, Fresno, Los Angeles, and the Inland Empire. Members met monthly via teleconference from July 2020 to February 2022 and were paid for their time and expertise. Meetings initially focused on building rapport, familiarizing members with the project objectives, and developing and adopting shared values and principles (Table 1). Later meetings focused on the activities described in Steps 2–4.

**Table 1:** Steering committee’s shared values and principles^1^

**Shared Values**
1. Prioritization and centering of the needs and experiences of BIPOC who have experienced pregnancy and/or childbirth and the community doulas who serve them 2. Commitment to expanding the knowledge on the value of doula care for families who need doula care and institutions in which they operate 3. Mutual respect of the knowledge and perspectives shared by all members 4. Fostering an environment of co-learning 5. Acknowledgment of each steering committee member’s wisdom and their valuable contributions 6. Shared responsibility and commitment to the project 7. Utilization of an asset-based framework, rather than a deficit-based framework
**Shared Principles**
1. Community doulas are aiding clients in a racist, structurally inequitable system; community doula care, alone, cannot eradicate poor birth outcomes for BIPOC. 2. Community doulas face many barriers to providing doula services, including barriers to obtaining a livable wage and/or certification. 3. Community doulas provide holistic care that cannot be measured solely by birth outcomes. 4. There is a general lack of knowledge among clients, public health practitioners, and healthcare providers, alike, regarding what community doula care is and what benefits they provide.

^1^ Adapted from the CRITICAL Membership Agreement/Memorandum of Understanding (Mohatt & Brenner, n.d.)

### Step 2: Conduct Needs Assessment to Understand Needs and Values around Research on Community Doula Care

In this step, we conducted a needs assessment to identify and understand stakeholders’ needs and values for research on community doula care. This was a particularly important step before engaging in future research because many community doulas and their clients are from groups that have a history of underrepresentation, exploitation, and exclusion in research (George et al., 2014).

We conducted semi-structured interviews with individuals from the seven stakeholder groups using an interview guide based on leadership team expertise and input from the Steering Committee. We used a purposive and snowball sampling strategy to recruit participants, leveraging the networks of our leadership team and Steering Committee. The Committee for the Protection of Human Subjects at the University of California, Berkeley approved the study protocol.

Interviews occurred from November 2020 to May 2021 and explored participants’ knowledge of, value for, and perceptions of gaps in research on community doula care, feelings about how research is typically conducted and should be used, and their willingness to participate in research. We used a Rapid Assessment Process (RAP) to analyze the interview data. To develop preliminary, actionable findings, RAP utilizes iterative analysis, additional data collection, and data triangulation (Beebe, 2008; Hamilton, 2013).

We conducted 29 interviews across the seven stakeholder groups: clinicians (*n* = 8), public health professionals (*n* = 8), doulas (*n* = 4), former doula clients (*n* = 3), researchers (*n* = 3), and policymakers or advocates (*n* = 3). Some participants represented multiple stakeholder types (e.g. a former doula client who works in advocacy). All but one participant lived and worked in California.

Overall, participants felt that research on community doula care was important and could be used positively to increase access to doula care by informing policy changes and helping doulas be more welcomed in medical settings (Table 2). Participants identified gaps in doula-related research on appropriate compensation models for doula care, interactions between doulas and hospital care teams, and racism in hospital settings and in the doula profession.

**Table 2:** Needs assessment findings

Domain	Findings
**Knowledge of** **doula care**	• Many participants reported that there is a general lack of knowledge around what doula care is and how it benefits birthing people and their families. • Some medical providers knew about doulas only in the context of labor and birth but did not know what doulas did with clients outside of that setting.
**Knowledge of community doula care**	• Participants familiar with community doulas described them as being members of the community they serve. Participants said community doulas typically serve anyone who needs support, regardless of their ability to pay. • Multiple participants talked about racial and/or cultural concordance in their descriptions of community doulas. • Having a broader scope of work than a traditional doula ^1^, including providing connections to resources, was described as a hallmark of community doula care.
**Value of research**	• While many participants knew about research on doula care, most did not know of any research about community doula care, specifically. • Most participants felt that research on community doula care was important and useful. • A few participants clarified that research should not be needed to “justify” doula care, as they felt that this type of care should not need justification. • Participants said research on community doula care could be used to: 1) increase access to doula care by informing funding decisions and policy changes; 2) help doulas be more welcomed in medical settings; and 3) provide education for various audiences around what doula care is. • One participant expressed a fear that increased research and attention to doula care may lead to regulation of the profession, which may make it inaccessible to community doulas. • Participants reported research is useful when: 1) it provides rationale for funding and increased access to doula care; 2) it centers the voices of a diverse range of doulas and clients, particularly Black and other BIPOC communities; and 3) findings are disseminated widely. • Participants reported research is *not* useful when: 1) it perpetuates negative racial stereotypes, narratives, and dangerous biases; 2) it does not center the voices of BIPOC doulas and clients; and 3) researchers are not inclusive and transparent.
**Gaps in research**	• Several participants were interested in payment and compensation models for doula care, including Medi-Cal coverage of doula services. • Various participants thought there should be more research on interactions between doulas and care teams in hospital settings and how to best integrate doulas into these care teams. • A few participants wanted to see more studies about doula support for non-birthing partners. • Generally, participants reported there weren’t enough studies that examined racism in hospital settings and racism in the doula profession. • Participants’ perspectives on the availability of research on birth outcomes were mixed; some felt that there was enough of this research available, while others felt birth outcomes could be studied further.
**Concerns about how research is conducted**	Participants’ reported their concerns about how research is typically conducted: • Research being exploitative or extractive • Biases affecting studies and results • Whether findings are adequately disseminated to communities • Research being conducted with a Western, white supremacist framework • Research on Black communities not being led by Black people
**How research should be conducted**	Participants’ reported their suggestions for how future research studies should be conducted: • Utilize focus groups, which give clients opportunities to share their experiences and allow for community healing • Incorporate more community members into research from the start • Create opportunities for Black people to be involved in research in a leadership capacity • Create funding opportunities for Black people to conduct their own research • Be transparent about who is conducting the research
**Research participation**	• Most participants expressed being open to participating in research about community doula care. Reasons for willingness to participate in research included: 1) making their voice heard and providing new perspectives; and 2) contributing to the knowledge base. • Some participants said they would want more information about the research before choosing to participate. • Several participants shared the belief that their peers would be open to participating in research. However, some participants described reasons why their peers might be hesitant: 1) concerns about research participants being compensated fairly for their time; 2) who is conducting the research; 3) who is included in the research; and 4) overall distrust in research as an institution.

^1^ In this context, “traditional doula” refers to a private, fee-based doula

Interviews surfaced concerns regarding research. One participant expressed a fear that increased research and attention to doula care may lead to regulation of the profession, which may make it inaccessible to community doulas. For example, some states require doulas be certified by specific doula organizations to enroll as Medicaid providers. However, obtaining certification can be costly and does not necessarily indicate relevance or competency in working with families at the intersection of racism and/or disadvantaging social determinants. Other participants expressed concerns about research being exploitative or extractive, biases affecting studies and results, and findings not being adequately disseminated to communities.

Participants suggested incorporating more community members into research from the start, creating funding opportunities for Black people to conduct their own research, and being transparent about who is conducting research. Participants also shared preferred research methodologies, including community-engaged research and qualitative methods such as focus groups, which give doula clients opportunities to share their experiences and can allow for community healing.

### Step 3: Identify a Shared Research Agenda Related to Community Doula Care

Informed by important contextual information about *how* future research on community doula care should be conducted, we next focused on identifying specific research topics and questions to be prioritized. We adapted the Research Prioritization by Affected Communities (RPAC) protocol, which aims to initiate patient and public involvement in setting research agendas, to guide this process (Franck et al., 2018). We engaged the Steering Committee in three virtual RPAC sessions from July 2021 to September 2021, with intermediate activities between meetings.

**Fig. 1:**
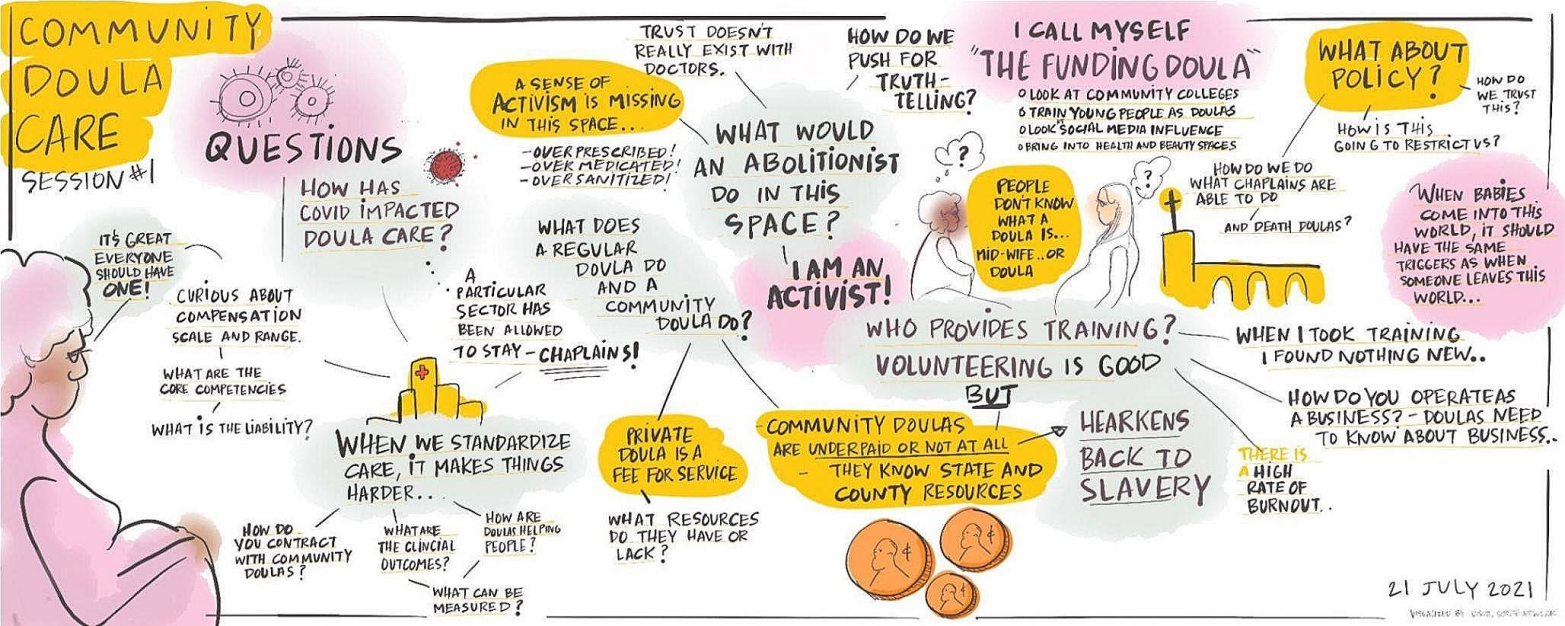
Graphic recording of the Steering Committee’s discussion about community doula care during the first RPAC session by visual scribe Ashanti Gardner

Session 1 involved a guided discussion to generate potential research questions about community doula care, which were grouped into themes and then reviewed carefully in session 2. The first and second sessions generated a total of 178 questions. A visual scribe recorded the first two sessions by drawing illustrations and writing words in real time. This process created a visual representation of each session that we shared with the Steering Committee to facilitate discussion and to serve as a reminder of commonly discussed topics (Fig. 1). Prior to session 3, the Steering Committee voted on questions most important to include in a research agenda about community doula care and, in the final session, confirmed the prioritized list of questions.

The final list of 21 priority research questions (Table 3) represented 10 themes: (1) scope of community doula care; (2) awareness of doula care; (3) doula training and certification; (4) workforce development; (5) policy and standardization; (6) ethics; (7) metrics, outcomes, and mechanisms; (8) COVID-19; (9) compensation and funding; and (10) integration and interactions with health systems. Notably, many questions focused on the implementation and sustainability of community-based doula programs and identification of appropriate metrics/measures of successes.

**Table 3:** Priority questions identified by steering committee through RPAC process

Theme	Research questions
**Scope of community doula care**	• What is the difference between the scope of work of a community vs traditional doula? ^1^ • What are the core competencies that every doula should have?
**Awareness of doula care**	• How do we educate clinicians and health systems about doula care?
**Doula trainings and certification**	• How are most community doulas trained and by whom? • Do doulas need continuing education courses? If so, on which topics and how often do they need to take the courses?
**Workforce development**	• How do we envision and reimagine the birthing workforce that people want, desire, and deserve? • How can we give doulas the professional development they need (e.g. financial literacy, capacity building, ins and outs of operating a business, referrals, and consultations)? • How do we provide pathways to birth work, including opportunities for youth?
**Ethics**	• What do equitable/fair/just labor conditions look like for community doulas?
**Compensation and funding**	• What is the range of compensation for community doula care? • What are appropriate rates for Medi-Cal reimbursement for doulas? • How can we redesign the social safety net and have doulas be a central part of it? • Does funding for community doula programs include the true cost of programming (e.g. legal counsel, insurance for the organizations and the doulas, marketing, evaluation, and administrative support needed to run the programs)? • What are the different funding models for community doula organizations? • How do we establish reimbursement models that support doula work as a profession and not a side job?
**Metrics, outcomes, and mechanisms**	• What are the metrics we should use to understand the impact/efficacy of community doula care?
**COVID-19**	• What did we learn from the COVID-19 pandemic regarding community doula care that we can leverage to make it more sustainable?
**Policy and standardization**	• How will government involvement (Medi-Cal reimbursement) impact the community doula space? • How can we build trust and foster respectful conversations with doulas who are resistant to regulations?
**Integration and interactions with health systems**	• How can doulas and clinical teams have mutually respectful relationships that support clients? • How can health insurance companies contract with doulas in a human-centered and collaborative way that works in the long run?

^1^ In this context, “traditional doula” refers to a private, fee-based doula

### Step 4: Increase Capacity among Community Doulas to Engage in Research on Community Doula Care

The final step of the project focused on a training to build skills relevant to answering research questions developed in step 3. With guidance from the Steering Committee, we decided to focus our training efforts on community doulas, as they are increasingly asked to collect data for evaluations of community-based doula programs and participate in research about their work. However, they are not often invited to develop evaluation methods or informed about research goals.

During the virtual, two-hour workshop, we disseminated the needs assessment and research prioritization findings, introduced research concepts, and provided a space for community doulas and other stakeholders to discuss the relevance and impact of research on their work. In a didactic portion of the workshop, we presented information about the research process and common research types, including community-engaged research (Key et al., 2019) and comparative effectiveness research (Sox, 2010). Project leaders, including doula leaders and a researcher, also shared their experiences with partnered research during a panel discussion.

## Assessment

Below we discuss successes, challenges, and lessons learned while carrying out this project that are relevant for future research on doula care.

### Research Prioritization Identified Important Understudied Questions that will Need to be Further Developed

While the list of priority research questions lays out important areas of inquiry for further exploration, we recognize that it is a starting point, and researchers will likely need to further develop the questions to be clearly defined, specific, and focused, which is important for successful research projects. By design, most Steering Committee members did not have a background conducting research, which we believe was a strength of the project. Our goal was not to produce perfected research questions, but, rather, to identify questions deemed to have shared importance across stakeholder groups and allow for partnered research.

We also hope that our list of questions can influence funders’ research priorities. Notably, none of the priority questions focused on clinical birth outcomes. Our Steering Committee and some needs assessment participants recognized that there is existing evidence that doula care improves clinical outcomes and, as such, felt there was a need for more research on the impact of community doulas on clients’ overall well-being, including social support, and birthing experiences. Further, funders, researchers, doulas, and community members measure success differently. As such, shared metrics must be holistically defined by those most affected.

### Challenges and Benefits of Evolving Policy Related to Expansion of Doula Services in California

About halfway through the project, the 2021–2022 California state budget provided funding to add doula services as a covered Medi-Cal benefit (Department of Health Care Services, n.d.). As a result, our Steering Committee meetings evolved to serve not only as a time to discuss research-related matters, but a gathering space for members to engage in conversations related to upcoming policy. In many regards, this was a positive outcome of our capacity-building process and reflected the strong relationships members developed. At the same time, it was challenging to maintain focus on research when there were pressing policy considerations at hand. Many members were aware of the challenges that occurred with Medicaid coverage of doula care in other states and used our meetings to discuss how implementation in California could avoid these pitfalls.

### Overlapping Findings Provide Direction for Future Research on Doula Care

Our findings provide direction for those interested in conducting research on doula care, as well as policymakers and funders. We observed overlapping findings across project phases, which indicates data triangulation and, thus, increases the validity of our results (Carter et al., 2014). First, regarding future research topics, we found in both the needs assessment and the research prioritization that there is a need to explore dignified compensation models for doula care and identify ways to build mutually respectful relationships between doulas and clinicians. Second, our needs assessment findings offer insight into acceptable research practices, which we also discussed during Steering Committee meetings and the research workshop. There was a desire for researchers to engage clients, community doulas, community-based doula organizations, and other stakeholders early in the research process and to center the voices of Black, Indigenous, and people of color (BIPOC) community members, who are most impacted by the maternal health crisis. This finding suggests that researchers should utilize community-engaged approaches for future research on doula care. Study designs like randomized controlled trials, in which one group receives the intervention and the other does not, would not be acceptable. Given existing evidence demonstrating the benefits of doula care, stakeholders perceived research involving withholding doula care from pregnant and birthing people as unethical. Lastly, throughout the project, stakeholders expressed a preference for qualitative data collection methods, which can create opportunities for storytelling and community healing. Stakeholders also noted that asking doulas to provide documentation and collect data for research purposes can be overly burdensome, especially when funders, including health plans, and researchers do not seek doula input.

### Need to Increase Public Awareness about Community Doula Care

A key observation from Steering Committee members, needs assessment participants, and workshop attendees was a need to increase awareness about the community doula care model across the general public, health care providers, and health systems. As California rolls out its Medi-Cal doula benefit, increasing awareness will be a critical step to ensuring pregnant people utilize the benefit, if desired, while developing more support for doulas in clinical environments.

## Limitations

While we believe our approach and findings have broad implications, we engaged stakeholders from a single state that was advancing Medicaid coverage of doula care. Additionally, although we attempted to engage all identified stakeholder groups, clients ended up having less representation on our Steering Committee due to some dropping out, potentially due to meetings occurring during working hours. Engagement, with respect to attendance at project meetings and events, was challenging overall due to the demands of the COVID-19 pandemic on our stakeholders, who mostly worked in health care or public health.

## Conclusion

The increasing utilization of doula care, and specifically community-based models of doula care, to address inequities in maternal and infant health provides an opportunity to think critically about research. The findings of our stakeholder-engaged process provide a roadmap that will lead to equity-oriented research centering clients, doulas, and their communities. Collaboration is critical for future research on doula care; our project findings suggest there is a welcome opportunity for partnered research if approached carefully and thoughtfully.

## Data Availability

The data generated from this study are not publicly available.
